# Identification and Characterization of *Limosilactobacillus reuteri* Strain S3 Isolated from Chicken and Its Protective Efficacy Against Avian Coccidiosis

**DOI:** 10.3390/microorganisms14081659

**Published:** 2026-07-29

**Authors:** Nianyu Xue, Qianqian Feng, Dandan Liu, Weimin Cai, Yuxin Zhou, Zhaofeng Hou, Jinjun Xu, Jianping Tao

**Affiliations:** 1 College of Veterinary Medicine, Yangzhou University, Yangzhou 225009, China; yznianyuxue@126.com (N.X.); qianqianfeng98@163.com (Q.F.); ddliu@yzu.edu.cn (D.L.); wmcaii@126.com (W.C.); zhouyuxin0613@163.com (Y.Z.); zfhou@yzu.edu.cn (Z.H.); jjxu@yzu.edu.cn (J.X.); 2 Jiangsu Co-Innovation Center for Prevention and Control of Important Animal Infectious Diseases and Zoonoses, Yangzhou University, Yangzhou 225009, China

**Keywords:** *Limosilactobacillus reuteri*, identification, probiotic properties, anticoccidial effect, chickens

## Abstract

*Limosilactobacillus reuteri* is one of the common lactic acid bacteria in the chicken gastrointestinal tract. Our previous study revealed that *L. reuteri* abundance declined in White Leghorn chickens following infection with *Eimeria tenella*, *E. maxima*, and *E. necatrix*, suggesting a potential role of this species in gut homeostasis during coccidiosis. In this study, eight *L. reuteri* strains were isolated from the intestinal tract of White Leghorn chickens. Strain S3 was selected as the most promising candidate after screening for acid and bile tolerance, adhesion ability, antagonism against pathogens. Oral administration of S3 strain at dose of 4 × 10^7^ CFU/bird, 1.2 × 10^8^ CFU/bird or 2 × 10^8^ CFU/bird for 18 days improved average daily gain (ADG) in both White Leghorn chickens and Suqin laying hens. The ADG value in groups treated with 4 × 10^7^ CFU/bird was significantly higher compared to that in the control group (*p* < 0.05). This treatment did not cause adverse effects on hepatic or renal function, but significantly increased the thymus index in both breeds and the levels of total protein and globulin in Suqin laying hens (*p* < 0.05). S3 strain also modulated gut microbiota composition in White Leghorn chickens, promoting beneficial lactobacilli colonization in the jejunum and achieving persistent colonization in the cecum. S3 strain selectively increased the proportion of CD3^+^CD4^+^ T lymphocytes in peripheral blood of Suqin laying hens. The anticoccidial efficacy of S3 strain was evaluated in Suqin laying hens challenged with *E. tenella*, *E. maxima*, and *E. necatrix*. The results showed that S3 strain alleviated clinical signs, reduced lesion scores, decreased oocyst output, and attenuated body weight loss. Anticoccidial index (ACI) values were 130.67, 148.06, and 163.40 for *E. tenella*, *E. maxima*, and *E. necatrix* infection, respectively. These results suggest that *L. reuteri* S3 is a safe chicken-derived probiotic with immunomodulatory and anticoccidial properties, and may serve as a feed additive for coccidiosis control in poultry production.

## 1. Introduction

The poultry industry is a major source of animal protein, with global production exceeding 70 billion chickens annually and accounting for approximately 40% of total meat output [1,2]. Poultry production is threatened by a variety of diseases. Among these, coccidiosis, caused by *Eimeria* spp., has been ranked as the top disease concern by members of the Association of Veterinarians in Broiler Production (AVBP) in annual surveys [3], with estimated annual losses exceeding £10.36 billion [4]. Current control of coccidiosis relies on anticoccidial drugs and live vaccines [3]. However, the widespread emergence of drug-resistant *Eimeria* strains, along with the risks of virulence reversion and pathogen dissemination associated with live vaccines, highlights the urgent need for safe and effective alternative control strategies [5,6].

Probiotics, as live nonpathogenic microorganisms, have the potential to maintain a normal gut microbiota through diverse modes of action, including competitive exclusion, production of antibacterial compounds, stimulation of the immune system, enhancement of gut barrier integrity and reduction in the severity of intestinal lesions. They are widely used in the poultry industry as natural alternatives to traditional chemotherapeutic drugs [7,8,9], with some probiotics exhibiting anticoccidial properties, such as *Bacillus subtilis*, *Saccharomyces cerevisiae*, *Enterococcus* spp., *Bifidobacterium* spp., *Pediococcus* spp., and *Lactobacillus* spp. [8,10,11,12,13,14]. Among these probiotics, some are derived from non-poultry or environmental sources [7]. Generally, the efficacy of probiotics exhibits high host specificity, as host-specific bacteria possess a natural advantage in colonizing the specialized avian digestive tract [15,16]. Therefore, poultry-derived probiotics may be far more effective against poultry coccidiosis [17,18].

Chickens are currently known to be susceptible to seven *Eimeria* species, with *E. tenella*, *E. necatrix* and *E. maxima* demonstrating the greatest pathogenicity and highest clinical risk [19,20,21]. Infection with these three *Eimeria* species disrupts intestinal integrity and microbiota balance, resulting in impaired nutrient absorption, poor growth performance, increased mortality, and heightened vulnerability to secondary infections in chickens [22,23,24,25]. All these three *Eimeria* infections lead to a reduction in gut commensal bacteria, especially lactic acid bacteria, in White Leghorn chickens. *Limosilactobacillus reuteri* (formerly *Lactobacillus reuteri*) consistently shows the greatest decline across all infections, suggesting that *L. reuteri* may play a role in maintaining gut homeostasis in chickens [26].

*L. reuteri* is one of the common lactic acid bacteria in the chicken gut and helps to maintain a healthy digestive system, thereby promoting the growth performance and overall health of poultry [17]. Certain strains of *L. reuteri* can produce antimicrobial substances such as lactic acid, acetic acid, ethanol, reutericyclin, and reuterin, which combat bacterial infections in the gastrointestinal tract, including those caused by *E. coli* and *Salmonella* [9]. It has also been reported to modulate host immune responses and strengthen intestinal barrier function [27,28]. A current study showed that *L. reuteri*, alone or in combination with berry pomace, has the potential to improve broiler growth performance, bone mineral, and body fat contents in broilers infected with mixed *E. acervulina*, *E. maxima* and *E. tenella* [29]. Supplementation with *L. reuteri* had a significant impact on oocyst shedding of *E. tenella* in broilers. Both *L. reuteri* and *Bacillus subtilis* supplementation resulted in higher villous height. Additionally, *L. reuteri* improved *Lactobacillus* and *Bifidobacterium* populations in both the ileum and caecum [30]. Different strains of *L. reuteri* have evolved through natural selection in various hosts, endowing them with specialized capabilities [31,32]. Therefore, identifying and isolating native strains is a highly effective strategy for optimizing probiotic performance [33].

The present study aimed to select, identify, and evaluate host-derived *L. reuteri* strains isolated from chicken intestinal contents using a microbiota-informed screening strategy based on *Eimeria* infection-associated microbiota alterations. Functional and safety characteristics of these isolates were assessed to determine their suitability as probiotics. By considering strain-specific properties, including acid and bile tolerance, adhesion ability, antagonistic activity against pathogens, safety-related characteristics, and anticoccidial efficacy against *E. tenella*, *E. maxima*, and *E. necatrix*, this study provides insights into the development of effective host-derived probiotics specifically tailored for avian coccidiosis control.

## 2. Materials and Methods

### 2.1. Chickens, Parasites, and Bacterial Strains

One-day-old specific pathogen-free (SPF) White Leghorn chickens were obtained from Lihua Agricultural Technology Co., Ltd. (Ningbo, China). One-day-old Suqin laying chicks were obtained from Yangzhou Xianglong Poultry Industry Development Co., Ltd. (Yangzhou, China). Suqin laying hens are a Chinese green-shelled egg-laying breed. The chickens were raised under coccidia-free conditions in wire cages and provided a standard corn- and soybean-based diet purchased from Jiangsu Xietong Pharmaceutical Bio-Engineering Co., Ltd. (Nanjing, China). No antibiotics or anticoccidial agents were administered throughout the experiment. The Yangzhou strains of *E. tenella*, *E. maxima*, and *E. necatrix* used in this study were isolated by the Parasitology Research Laboratory at Yangzhou University, China, and confirmed through microscopic examination and sequence analysis of the internal transcribed spacer region of genomic DNA. These strains have been preserved in our laboratory [34,35,36]. *Clostridium perfringens* ATCC 13124, *Escherichia coli* ATCC 25922, and *Staphylococcus aureus* ATCC 25923 were purchased from Huankai Biotechnology Co., Ltd. (Zhaoqing, China). *Salmonella pullorum* was maintained in our laboratory. *Limosilactobacillus vaginalis* ATCC 55739 and *Lactobacillus plantarum* ATCC 8014 were purchased from Mingzhou Biotechnology Co., Ltd. (Ningbo, China).

### 2.2. Isolation and Identification of Chicken-Derived L. reuteri Strains

*L. reuteri* strains were isolated from SPF White Leghorn chickens (Lihua Agricultural Technology Co., Ltd., Ningbo, China). Three-week-old chickens were euthanized by CO_2_ asphyxiation followed by cervical dislocation. Under aseptic conditions, the intestinal tract was dissected and 0.3 g of jejunal and cecal contents were collected separately. Serial 10-fold dilutions (10^−1^ to 10^−7^) were prepared in PBS, and 100 μL aliquots from each dilution were spread onto MRS agar plates. Plates were incubated anaerobically at 37 °C for 24 h in an anaerobic jar (Mitsubishi Gas Chemical, Tokyo, Japan) using a commercial anaerobic gas-generating sachet (Hopebio, Qingdao, China). Colonies with typical *Lactobacillus*-like morphology were selected and purified by streak plating on MRS agar. Isolates were characterized by Gram staining and catalase testing. Gram-positive, catalase-negative isolates were subjected to 16S rRNA gene amplification using primers 7F (5′-AGAGTTTGATYMTGGCTCAG-3′) and 1510R (5′-ACGGYTACCTTGTTACGACTT-3′) [37]. PCR products were sequenced by Beijing Liuhe BGI Co., Ltd. (Beijing, China). Sequences were compared against the GenBank database using the NCBI BLAST online tool (https://blast.ncbi.nlm.nih.gov/, accessed on 23 March 2026), and a phylogenetic tree was constructed using the neighbor-joining method in MEGA 11.0 with 1000 bootstrap replicates [38]. The 16S rRNA gene sequences of the eight *L. reuteri* isolates have been deposited in the GenBank database under accession numbers PZ676512–PZ676519.

### 2.3. Tolerance to Simulated Gastrointestinal Fluid and Chicken Bile

Simulated gastric fluid (SGF) and simulated intestinal fluid (SIF) were prepared according to the Chinese Pharmacopeia (2010) [39]. Tolerance to SGF and SIF was assessed following Sornsenee et al. [40] with minor modifications. Overnight cultures (1 mL) were centrifuged at 6000 rpm for 5 min. The supernatant was discarded, and pellets were resuspended in 1 mL PBS to a concentration of 1 × 10^8^ CFU/mL. Bacterial suspensions (100 µL) were mixed with 900 µL of SGF or SIF and incubated anaerobically at 37 °C. Samples were taken at 0 and 3 h, serially diluted, and plated on MRS agar. Survival rate (%) = (N_3h_/N_0h_) × 100. Results were compared with two reference strains: *L. vaginalis* ATCC 55739 and *L. plantarum* ATCC 8014.

Bile tolerance was assessed following Xiang et al. [41], with modifications. Bacterial suspensions (1 mL; 1 × 10^8^ CFU/mL) were mixed with fresh chicken bile to achieve final concentrations of 40%, 60%, and 80% (*v*/*v*). Untreated suspensions served as controls. Mixtures were incubated anaerobically at 37 °C for 3 h, serially diluted, and plated on MRS agar for colony counting. Survival rates were calculated as the percentage of viable colonies relative to untreated controls. All assays were performed in triplicate.

### 2.4. Antibacterial Activity Against Pathogenic Bacteria

Antibacterial activity was evaluated using the Oxford cup method [42]. Overnight cultures in MRS broth were centrifuged at 6000 rpm for 5 min, and the supernatant was filtered through a 0.22 μm membrane to obtain cell-free supernatant (CFS). Indicator strains (*S. pullorum*, *E. coli* ATCC 25922, and *S. aureus* ATCC 25923) were cultured in LB broth at 37 °C for 18 h and adjusted to 1 × 10^8^ CFU/mL with PBS. Each indicator suspension (100 μL) was spread on LB agar plates. Sterile Oxford cups were placed on the surface, and 100 μL CFS was added to each cup. After incubation at 37 °C for 24 h, inhibition zone diameters were measured. All assays were performed in triplicate.

### 2.5. Adhesion Capacity to DF-1 Cells

Adhesion ability was assessed using DF-1 chicken embryo fibroblast cells (Pricella Biotechnology Co., Ltd., Wuhan, China). Cells were seeded at 1 × 10^5^ cells per well in 24-well plates containing sterile glass coverslips and incubated at 37 °C with 5% CO_2_ for 24 h. Monolayers were washed twice with PBS. Bacterial suspensions (1 × 10^8^ CFU/mL; 1 mL per well) were added in triplicate and incubated for 3 h. Coverslips were removed, washed three times with PBS to remove non-adherent bacteria, fixed with 4% paraformaldehyde for 30 min, air-dried, and stained with crystal violet. Adherent bacteria were observed under oil immersion microscopy, and the number of bacteria was counted in five randomly selected fields per coverslip.

### 2.6. Growth Kinetics of L. reuteri S3

The growth kinetics of *L. reuteri* S3 were determined by inoculating 10 μL (1%) overnight culture into 1 mL fresh MRS broth and incubating at 37 °C for 48 h. Absorbance at 600 nm (OD_600_) was measured every 2 h from 0 to 24 h and every 12 h from 24 to 48 h. Uninoculated MRS broth served as the blank control.

### 2.7. In-Vitro Safety Assessment

#### 2.7.1. Hemolytic Activity

Hemolytic activity of *L. reuteri* S3 was tested on Columbia blood agar containing 5% defibrinated sheep blood, with *C. perfringens* ATCC 13124 as the positive control. Plates were incubated at 37 °C for 48 h. Hemolysis was classified as β-hemolysis (complete lysis of red blood cells, resulting in a clear hemolysis ring surrounding the colony), α-hemolysis (partial hydrolysis of red blood cells, forming a greenish hemolysis ring around the colony), and γ-hemolysis (no hydrolysis of red blood cells and no hemolysis zone is observed around the colony) [43].

#### 2.7.2. Antibiotic Susceptibility

The antibiotic susceptibility of *L. reuteri* S3 was determined by the disk diffusion method [44]. Fifteen antibiotics were tested: erythromycin (15 μg), clarithromycin (15 μg), clindamycin (2 μg), chloramphenicol (30 μg), nitrofurantoin (300 μg), penicillin (10 μg), oxacillin (1 μg), tetracycline (30 μg), minocycline (30 μg), ciprofloxacin (5 μg), ofloxacin (5 μg), vancomycin (30 μg), polymyxin B (300 IU), co-trimoxazole (23.75/1.25 μg), and spectinomycin (100 μg). Antibiotic disks (Binhe Microorganism Reagent Co., Hangzhou, China) were placed on MRS agar plates inoculated with *L. reuteri* S3 (1 × 10^8^ CFU/mL). After incubation at 37 °C for 24 h, inhibition zone diameters were measured. Isolates were classified as susceptible (≥21 mm), intermediate (16–20 mm), or resistant (≤15 mm) [43].

### 2.8. In-Vivo Safety Evaluation

White Leghorn chickens and Suqin laying hens were used to assess the safety of *L. reuteri* S3. For each breed, 64 one-day-old chicks were adapted for 3 days and then randomly divided into four groups (*n* = 16) based on body weight. Three treatment groups received daily oral gavage of 200 µL containing *L. reuteri* at low (2 × 10^8^ CFU/mL), medium (6 × 10^8^ CFU/mL), and high (1 × 10^9^ CFU/mL) doses. The control group received 200 µL PBS. After administration continued for 18 consecutive days, the chickens were weighed to calculate the average daily gain (ADG). Then, 10 chickens from each group were used to evaluate the organ indices (organ weight/body weight). Three chickens were used to analyze serum biochemical parameters using a Mindray BS-420 automated chemistry analyzer (Mindray Bio-Medical Electronics Co., Ltd., Shenzhen, China). Another three chickens were continued to be raised for 7 days for analysis of gut microbiota composition.

### 2.9. Effect of L. reuteri S3 on Gut Microbiota

Following 18 days of administration and a 7-day withdrawal period, three chickens from each group were euthanized by CO_2_ asphyxiation followed by cervical dislocation. Jejunal and cecal contents were collected aseptically. Microbial DNA was extracted using the E.Z.N.A.^®^ Stool DNA Kit (Omega Bio-tek, Norcross, GA, USA). The V1–V9 region of the 16S rRNA gene was amplified using primers 27F (5′-AGRGTTYGATYMTGGCTCAG-3′) and 1492R (5′-RGYTACCTTGTTACGACTT-3′). SMRTbell libraries were constructed and sequenced on a PacBio Sequel IIe platform (Shanghai Biozeron Biotechnology Co., Ltd., Shanghai, China).

Raw reads were processed using SMRT Link (v9.0) to obtain CCS reads (minimum passes = 3, minimum predicted accuracy = 0.99), filtered for length (800–2500 bp) and quality, and chimeras were removed. OTUs were clustered at 98.65% similarity using UPARSE (v7.1, http://drive5.com/uparse/, accessed on 23 March 2026) and taxonomically assigned against the SILVA (SSU138) database at a 70% confidence threshold using RDP Classifier (https://sourceforge.net/projects/rdp-classifier/, accessed on 23 March 2026) [45]. Alpha diversity (Chao1 and Shannon indices) was calculated using Mothur (v1.21.1) [46]. Beta diversity was assessed by PCoA based on Bray–Curtis distances using the Vegan package (v2.6-2) in R (v4.0.2), and group differences were tested by PERMANOVA with 999 permutations [47,48].

Sample names were defined as follows: Uc represents the untreated control group; Low, Middle, and High represent the low-, medium-, and high-dose *L. reuteri* S3 supplementation groups, respectively; j and c indicate jejunal and cecal samples, respectively; and the number 7 indicates that samples were collected 7 days after cessation of *L. reuteri* S3 administration.

### 2.10. Anticoccidial Efficacy of L. reuteri S3 Against Three Eimeria Species

#### 2.10.1. Experimental Design

A total of 76 three-day-old Suqin laying hens with similar body weights were randomly assigned to seven groups: S3/*E. tenella*, S3/*E. maxima*, S3/*E. necatrix*, PBS/*E. tenella*, PBS/*E. maxima*, PBS/*E. necatrix*, and PBS/PBS (control group). Except for the S3/*E. tenella* and PBS/PBS group, each of which had 13 chickens with three chickens randomly selected for analysis of peripheral blood lymphocyte subsets prior to challenge, the remaining groups consisted of 10 chickens per group. From 4 to 22 days of age, S3/*E. tenella*, S3/*E. maxima*, and S3/*E. necatrix* groups received daily oral gavage of 200 µL *L. reuteri* S3 (2 × 10^8^ CFU/mL), while the remaining groups received equal volumes of PBS. At 14 days of age, S3/*E. tenella* and PBS/*E. tenella* groups were orally inoculated with 5 × 10^4^ sporulated oocysts of *E. tenella*; S3/*E. maxima* and PBS/*E. maxima* groups with 5 × 10^4^ sporulated oocysts of *E. maxima*; S3/*E. necatrix* and PBS/*E. necatrix* groups with 1.5 × 10^4^ sporulated oocysts of *E. necatrix*. The control group received an equal volume of PBS. The trial was terminated 8 days post challenge (on day 22).

#### 2.10.2. Anticoccidial Efficacy Assessment

All chickens were euthanized 8 days post challenge (on day 22). Protective efficacy was evaluated using multiple parameters, including number of bloody diarrhea (NBD), survival rate (SR), relative body weight gain (RWG), reduction in oocyst production (ROP), intestinal lesion score (LS), and anticoccidial index (ACI). Fecal droppings were examined visually for blood on days 4, 5, 6, 7, and 8 after inoculation at 12 h intervals. NBD was is the total number of bloody stools from each group. SR was calculated as the proportion of surviving birds in each group. Body weight gain (BWG) was measured from day 14 to day 22 (challenge period). RWG (%) was calculated as: (BWG of infection group/BWG of control group) × 100. Oocyst production (OP) was determined as described by Norton and Hein [49]. In the groups infected with *E. tenella* and *E. necatrix*, OP consisted of oocysts from feces and cecal contents. ROP (%) was calculated as: [(OP in PBS/infection group − OP in S3/infection group)/OP in PBS/infection group] × 100. LS was assessed on day 22 using a 0 to 4 scale as described by Johnson and Reid [50]. ACI calculated as follows: [(RWG + SR) − (Lesion index + Oocyst index)]. Lesion index (LI) = mean LS × 10. Oocyst index (OI) was assigned based on the ratio of oocyst production in S3/infection group to that in PBS/infection group: 0~1% = 0, 1~25% = 5, 26~50% = 10, 51~75% = 20, and 76~100% = 40. ACI values below 120 indicate no anticoccidial activity, 120–140 indicate minimal effectiveness, 140–160 represent moderate efficacy, 160–180 indicate good efficacy, and values exceeding 180 are considered excellent [51].

### 2.11. Flow Cytometry Analysis of Peripheral Blood Lymphocyte Subsets

At 14 days of age, prior to challenge, three chickens were randomly selected from the S3/*E. tenella* and the control group, and 2 mL of blood per bird was collected into anticoagulant tubes. Blood was diluted 1:1 with diluent and layered onto peripheral blood lymphocyte separation medium (Solarbio, Beijing, China) in 15 mL centrifuge tubes for lymphocyte isolation. Lymphocytes were counted and adjusted to 1 × 10^7^ cells/mL. Aliquots of 100 µL were incubated with mouse anti-chicken CD3, CD4, and CD8 antibodies (Southern Biotech, Birmingham, AL, USA) at 4 °C in the dark for 30 min, washed twice with PBS, and analyzed on a CytoFLEX flow cytometer (Beckman Coulter, Brea, CA, USA). Peripheral blood lymphocytes were first gated based on their forward scatter (FSC) and side scatter (SSC) characteristics. CD3^+^ T lymphocytes were subsequently identified, and the proportions of CD3^+^CD4^+^ and CD3^+^CD8^+^ T-cell subsets were analyzed within the CD3^+^ population.

### 2.12. Statistical Analysis

Data are expressed as mean ± SD. Statistical analysis was performed using SPSS 23.0 (IBM Corp., Armonk, NY, USA). Multiple group comparisons were analyzed by one-way ANOVA followed by Duncan’s multiple range test, and comparisons between two groups were performed using Student’s *t*-test. *p* < 0.05 was considered significant. Graphs were generated using GraphPad Prism 9 (GraphPad Software Inc., San Diego, CA, USA).

## 3. Results

### 3.1. Isolation and Identification of L. reuteri

**Figure 1:**
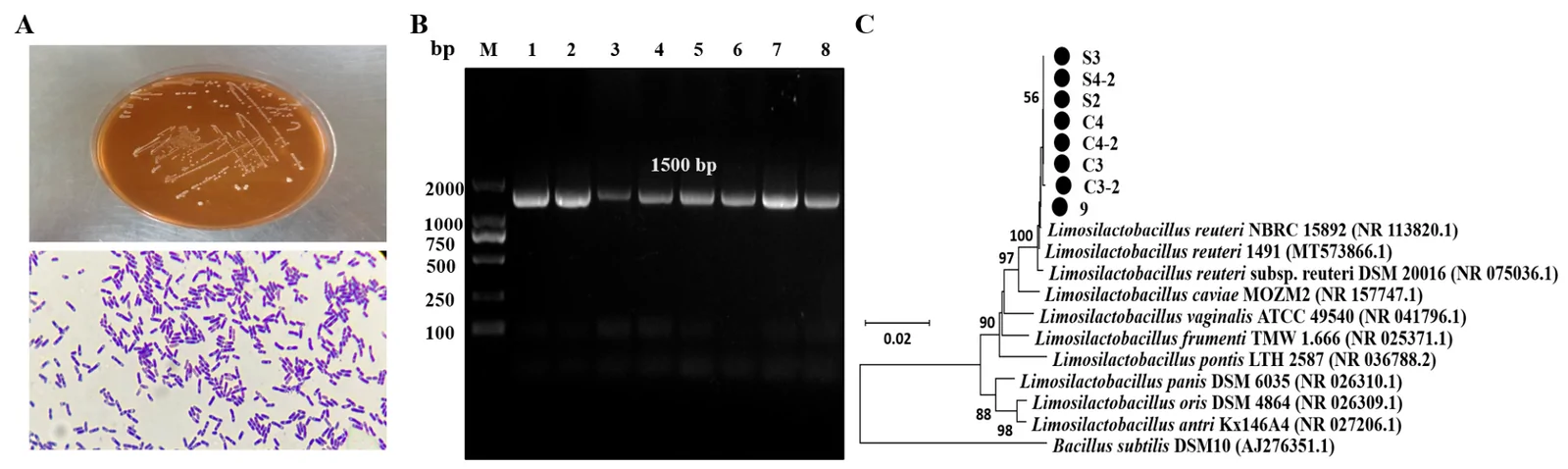
Isolation and identification of *L. reuteri* strains. (**A**) Colony morphology on MRS agar and Gram staining. (**B**) 16S rRNA gene PCR amplification products. (**C**) Phylogenetic tree based on 16S rRNA gene sequences constructed using the neighbor-joining method with 1000 bootstrap replicates.

Eight *L. reuteri* strains were isolated from the jejunum and cecum of White Leghorn chickens. Colonies on MRS agar were medium-sized, off-white, round, with slightly raised and smooth surfaces (Figure 1A). Gram staining showed blue-purple short rods (Figure 1A). 16S rRNA gene amplification yielded approximately 1500 bp fragments (Figure 1B). BLAST analysis indicated that all eight isolates shared >99% sequence similarity with *L. reuteri* reference strains in GenBank. Phylogenetic analysis confirmed that these isolates clustered within the *L. reuteri* clade (Figure 1C). Subsequently, a stepwise screening was used to select *L. reuteri* strains with superior probiotic potential.

### 3.2. Screening of L. reuteri Strains with Superior Probiotic Potential

**Figure 2:**
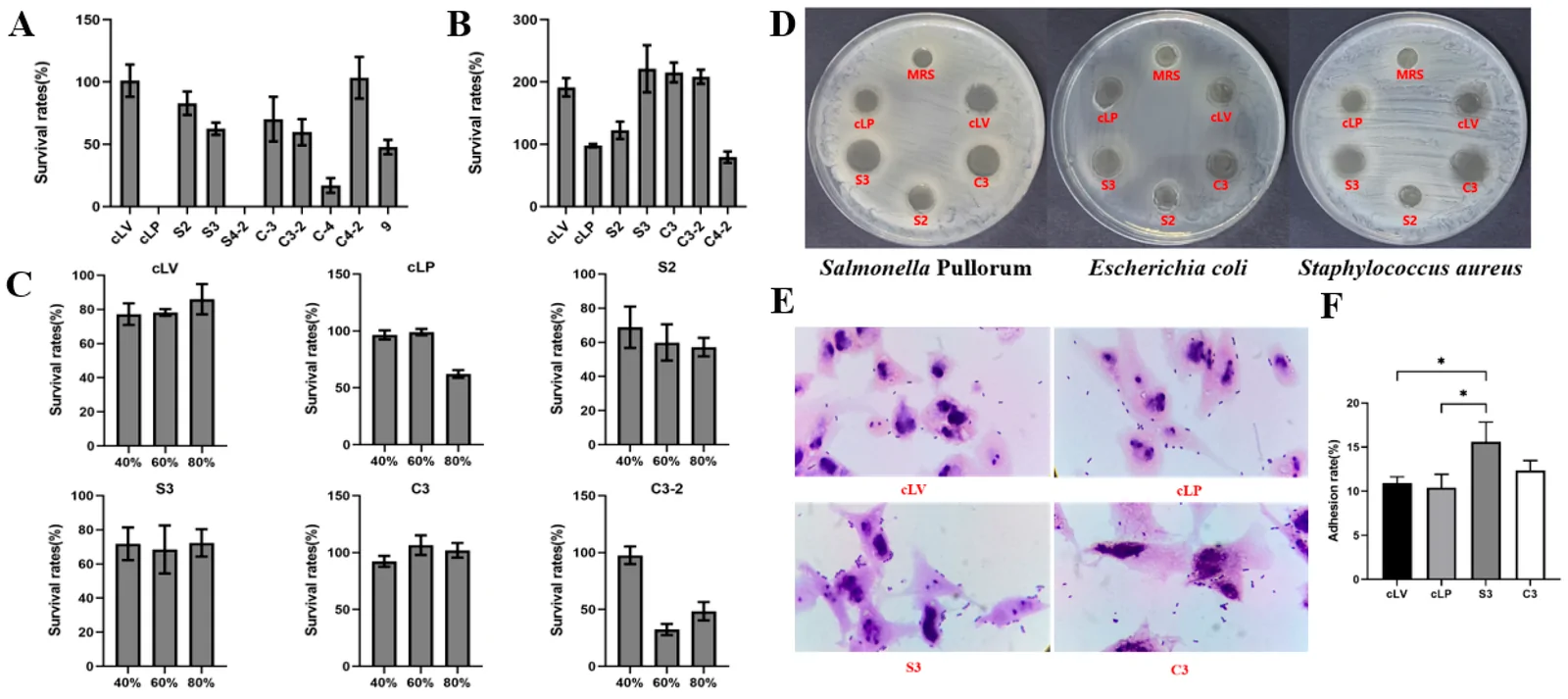
Screening of probiotic properties of *L. reuteri* isolates. Survival rates in simulated gastric fluid (**A**), and simulated intestinal fluid (**B**). (**C**) Survival rates in fresh chicken bile at different concentrations. (**D**) Inhibitory effects against *S. pullorum*, *E. coli*, and *S. aureus*. (**E**) Microscopic observation of *L. reuteri* adhesion to DF-1 cells (oil immersion, ×1000). (**F**) Adhesion counts of each strain to DF-1 cells. *, *p* < 0.05.

*Resistance of the strains to simulated SGF conditions*: Survival rates of the eight isolates were evaluated under SGF conditions. Based on the results, the viability of five strains (S2, S3, C3, C3–2 and C4–2) was above 50% under SGF conditions (Figure 2A).

*Resistance of the strains to simulated SIF conditions*: The survival rates of the five isolates were further evaluated under SIF conditions. Based on the results of their resistance to simulated GIT conditions, the viability of four strains (S2, S3, C3, and C3–2) exceeded 120% under SIF conditions (Figure 2B).

*Resistance of the strains to fresh chicken bile*: Based on the above results, the survival rates of the four isolates were evaluated under fresh chicken bile conditions. The results showed that three isolates (S2, S3, and C3) maintained survival rates above 50% at all tested concentrations (Figure 2C).

*Antimicrobial activity*: The ability to inhibit the growth of pathogens is a favorable characteristic of probiotic bacteria. Based on the well-diffusion method, the diameters of the growth inhibition zones of the pathogens treated with the three isolated strains (S2, S3, and C3) were more than 9 mm (Table 1), indicating that they inhibited the growth of *S. pullorum*, *E. coli* and *S. aureus*, with the S3 and C3 strains showing stronger inhibition (Figure 2D).

*Adhesion ability*: The adhesion feature is essential in selecting probiotic candidates because it facilitates the proliferation of lactobacilli. In this research, DF-1 cells were used to mimic the intestinal mucosal epithelium. Both the S3 and C3 strains demonstrated strong adhesion to DF-1 cells, with the S3 strain exhibiting significantly higher adhesion than the reference strains cLV and cLP (*p* < 0.05) (Figure 2E,F).

Based on performance in gastrointestinal tolerance, antibacterial activity, and adhesion capacity, *L. reuteri* S3 was selected for subsequent studies.

### 3.3. Growth Characteristics of L. reuteri S3

**Figure 3:**
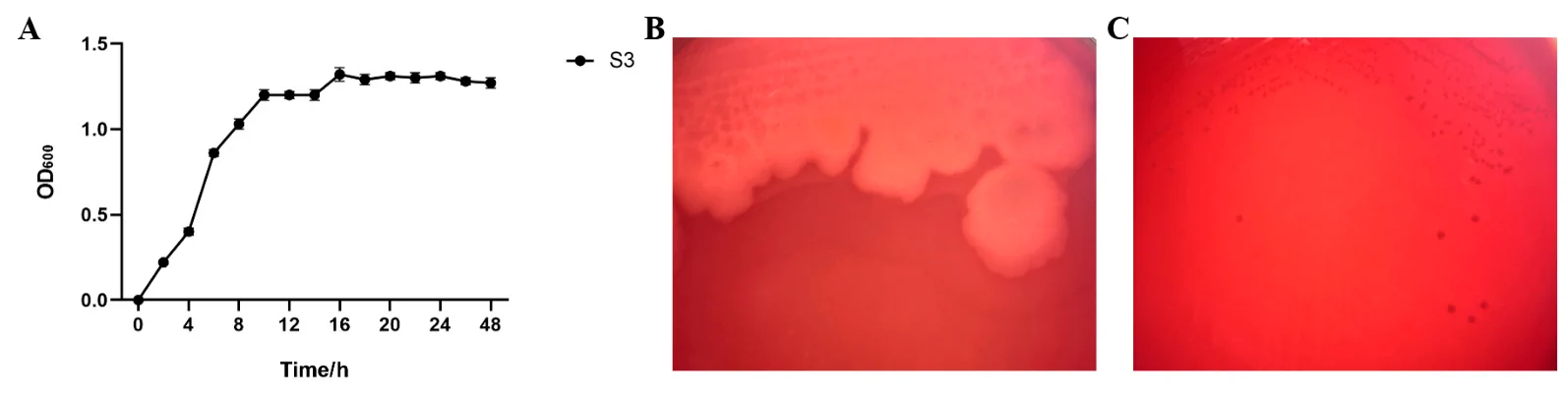
Growth curve (**A**) and hemolytic activity (**B**,**C**) of *L. reuteri* S3. (**B**) Positive control *C. perfringens* ATCC 13124 showing β-hemolysis. (**C**) *L. reuteri* S3 showing γ-hemolysis (no hemolysis).

After five consecutive generations, the growth curve of *L. reuteri* S3 was determined by measuring the value of OD600. The result showed that the OD_600_ value increased rapidly during the first 10 h, indicating exponential growth, and then plateaued, entering the stationary phase (Figure 3A).

### 3.4. In-Vitro Safety of L. reuteri S3

*Hemolytic activity*: The positive control strain *C. perfringens* ATCC 13124 produced clear β-hemolysis zones, while *L. reuteri* S3 showed no hemolysis (γ-hemolysis), confirming its non-hemolytic phenotype (Figure 3B,C).

*Antibiotic susceptibility*: The antibiotic susceptibility profile of *L. reuteri* S3 is summarized in Table 2. *L. reuteri* S3 was susceptible to erythromycin, clarithromycin, clindamycin, chloramphenicol, and penicillin, and resistant to nitrofurantoin, oxacillin, tetracycline, minocycline, ciprofloxacin, ofloxacin, vancomycin, polymyxin B, co-trimoxazole, and spectinomycin.

### 3.5. In-Vivo Safety of L. reuteri S3

**Figure 4:**
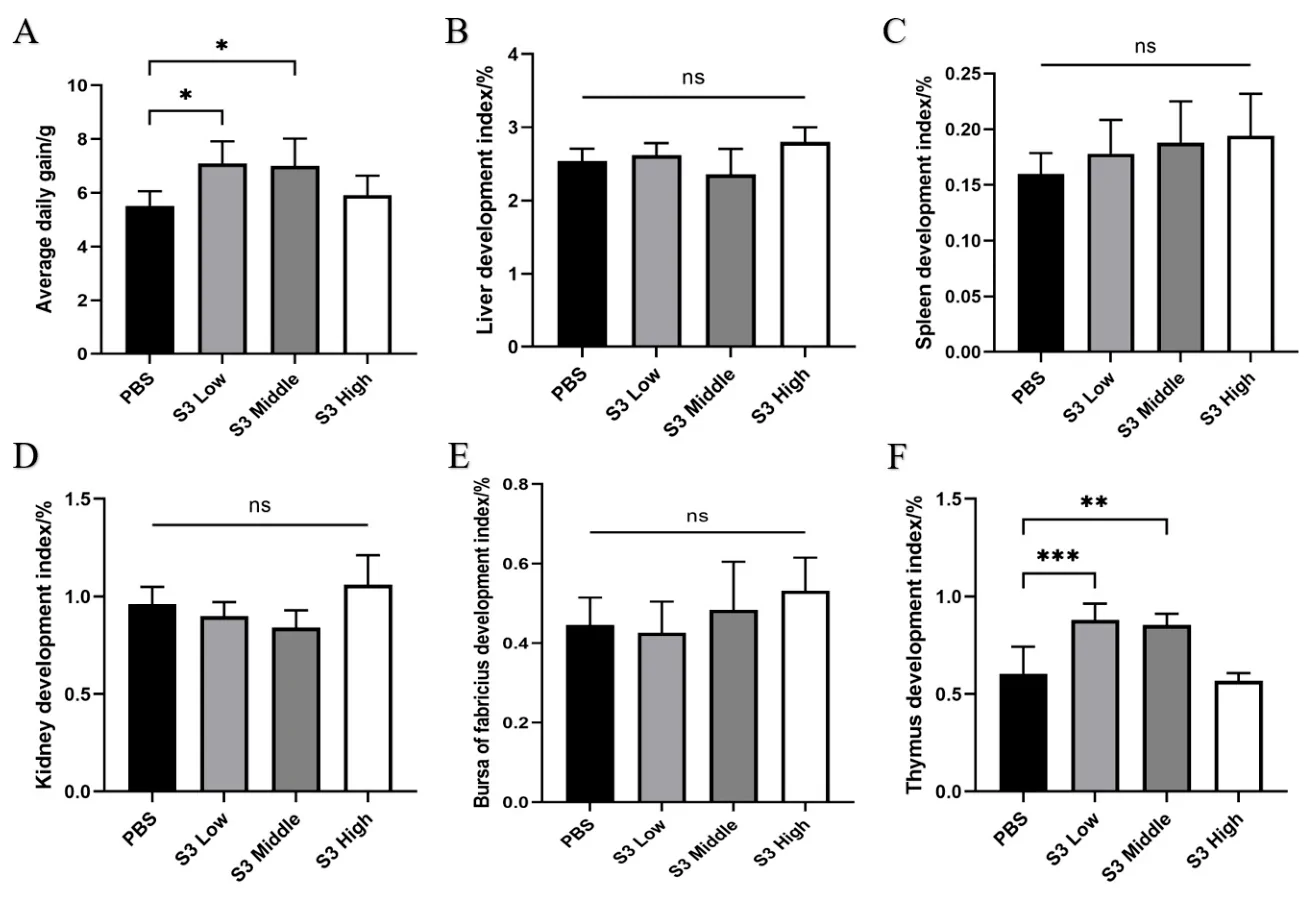
Effects of *L. reuteri* S3 on growth performance and organ indices in White Leghorn chickens. (**A**) Average daily gain. (**B**) Liver index. (**C**) Spleen index. (**D**) Kidney index. (**E**) Bursa of Fabricius index. (**F**) Thymus index. *, *p* < 0.05, **, *p* < 0.01, ***, *p* < 0.001, and ns, not significant.

**Figure 5:**
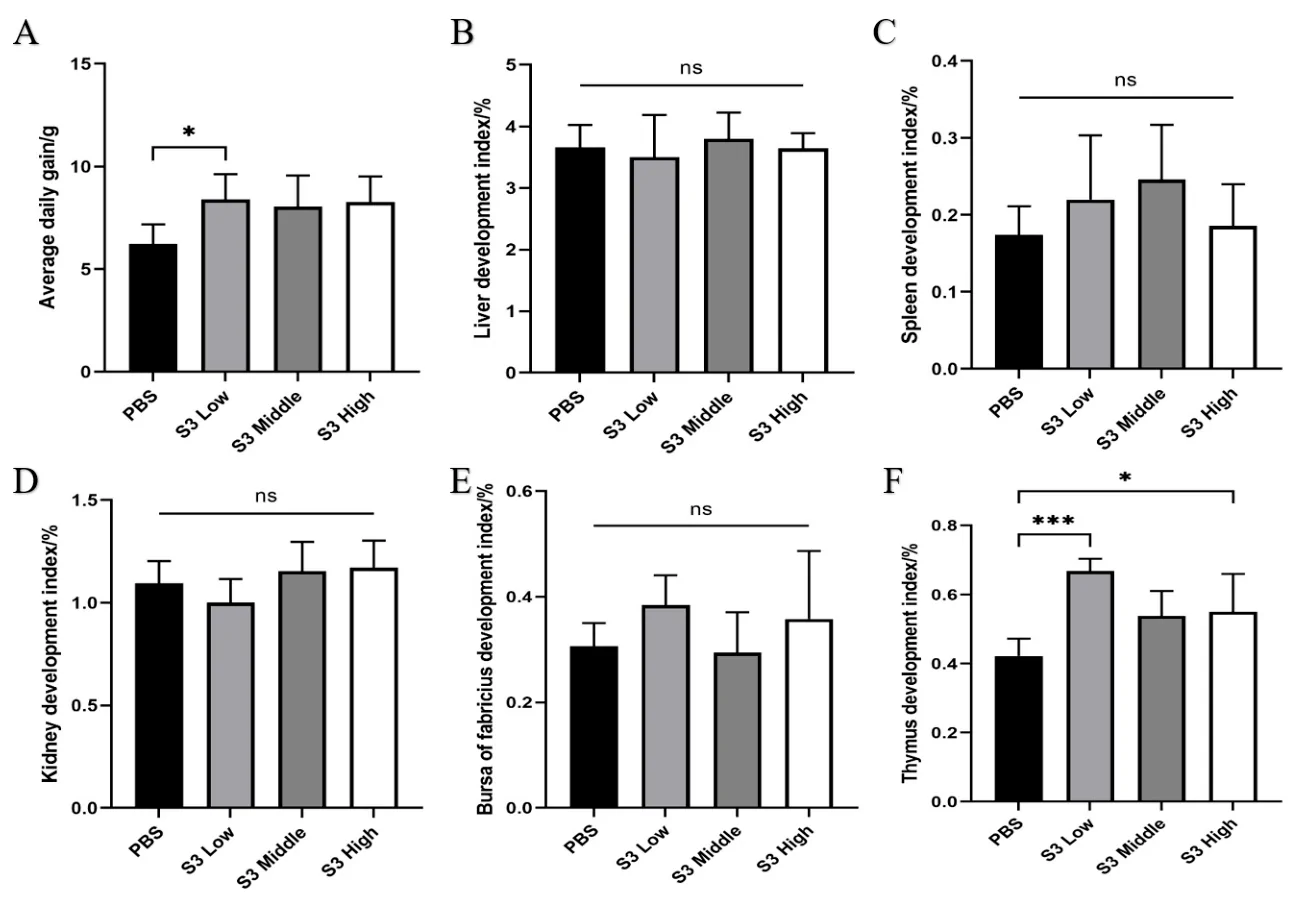
Effects of *L. reuteri* S3 on growth performance and organ indices in Suqin laying hens. (**A**) Average daily gain. (**B**) Liver index. (**C**) Spleen index. (**D**) Kidney index. (**E**) Bursa of Fabricius index. (**F**) Thymus index. *, *p* < 0.05, ***, *p* < 0.001, and ns, not significant.

*Growth performance and organ indices*: In White Leghorn chickens, ADG was significantly higher in the low- and medium-dose groups than in the control (*p* < 0.05) (Figure 4A). No significant differences were observed in the liver, spleen, kidney, and Bursa of Fabricius (Figure 4B–E), while the thymus index was significantly higher in the low- and medium-dose groups (*p* < 0.05) (Figure 4F). In Suqin laying hens, ADG was significantly higher in the low-dose group (*p* < 0.05) (Figure 5A). There were no significant differences in the indices of the liver, spleen, kidneys, and Bursa of Fabricius among groups (Figure 5B–E), while the thymus index was significantly higher in the low- and high-dose groups (*p* < 0.05) (Figure 5F).

*Serum biochemistry*: In White Leghorn chickens (Table 3), the values of alanine aminotransferase (ALT), aspartate aminotransferase (AST), total protein (TP), albumin (ALB), globulin (GLO), albumin-to-globulin ratio (A/G), urea, total cholesterol (TC), and triglycerides (TG) in all S3-treated groups showed no significant differences from the control group (*p* > 0.05), except for a significant increase in ALT in the high-dose group (*p* < 0.05).

In Suqin laying hens (Table 4), ALT, AST, ALB, A/G, urea, TC, and TG were not affected by S3 supplementation (*p* > 0.05), except for a significant increase in ALB and a significant decrease in TC in the high-dose group (*p* < 0.05). TP and GLO were significantly higher in all S3-treated groups (*p* < 0.05).

### 3.6. Effects of L. reuteri S3 Supplementation on the Gut Microbiota

**Figure 6:**
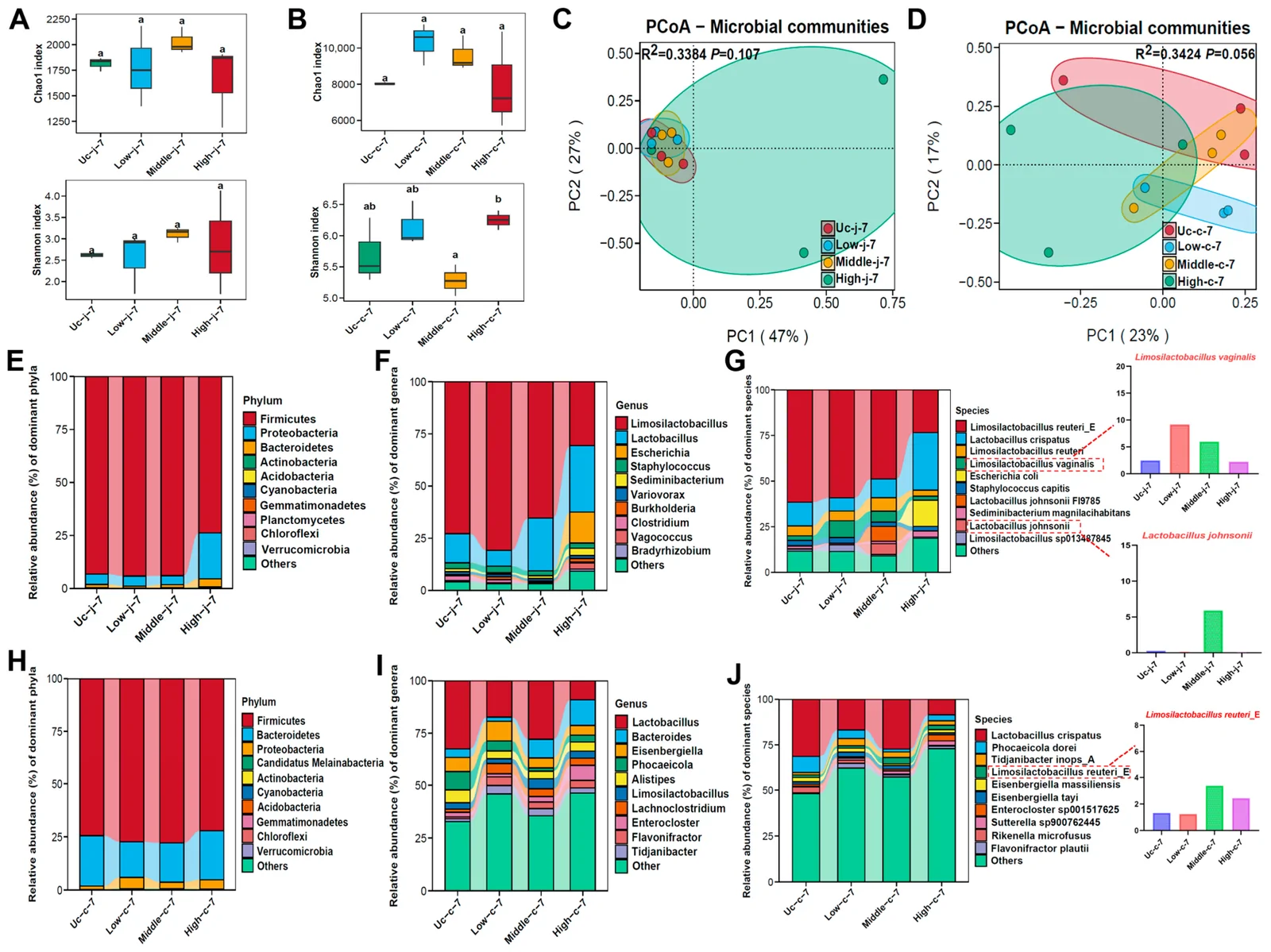
Effects of *L. reuteri* S3 supplementation on gut microbiota composition in White Leghorn chickens. (**A**) Alpha diversity (Chao1 and Shannon indices) of the jejunal microbiota. (**B**) Alpha diversity (Chao1 and Shannon indices) of the cecal microbiota. (**C**,**D**) Beta diversity (PCoA based on Bray–Curtis distances) of the jejunal (**C**) and cecal (**D**) microbiota. (**E**–**G**) Relative abundance of jejunal microbiota at the phylum (**E**), genus (**F**), and species (**G**) levels. (**H**–**J**) Relative abundance of cecal microbiota at the phylum (**H**), genus (**I**), and species (**J**) levels. Groups with different lowercase letters are significantly different (*p* < 0.05), whereas groups sharing one or more common letters are not significantly different.

*L. reuteri* S3 modulated gut microbiota composition in White Leghorn chickens (Figure 6). Chao1 and Shannon indices were higher in the medium-dose jejunal and low-dose cecal groups than the control (Figure 6A,B), PCoA based on Bray–Curtis distances showed partial separation between the treatment and the control groups in the jejunum (Figure 6C), with more pronounced separation observed in the cecum (Figure 6D).

In the jejunum, at the phylum level, the abundance of Firmicutes was increased in the low- and medium-dose groups, while that of Bacteroidetes and Proteobacteria was decreased. However, the abundance of Proteobacteria was elevated in the high-dose group (Figure 6E). At the genus level, *Limosilactobacillus* abundance increased, while *Lactobacillus* abundance decreased in the low-dose group. The medium-dose group exhibited the opposite pattern: *Limosilactobacillus* abundance decreased, whereas *Lactobacillus* abundances increased. In the high-dose group, *Limosilactobacillus* abundance decreased, while *Lactobacillus* and *Escherichia* abundances increased. *Clostridium* abundance decreased in all three groups (Figure 6F). At the species level, *L. vaginalis* abundance increased in the low-dose group; *L. reuteri, L. vaginalis*, and *L. johnsonii* abundances increased in the medium-dose group; *L. reuteri* abundance decreased, whereas *L. crispatus* and *E. coli* abundances increased in the high-dose group (Figure 6G).

In the cecum, Firmicutes and Proteobacteria abundances increased in the low- and middle-dose groups, whereas Bacteroidetes abundance decreased in all treatment groups (Figure 6H). At the genus level, *Lactobacillus*, *Phocaeicola*, and *Alistipes* abundances decreased, while *Tidjanibacter* abundance increased significantly in all treatment groups (Figure 6I). At the species level, *L. crispatus* and *Phocaeicola dorei* abundances decreased in all treatment groups, while *Flavonifractor plautii* abundance increased in the low-dose group. *L. reuteri* abundance was higher in the middle- and high-dose cecal groups than in the control (Figure 6J).

### 3.7. Anticoccidial Efficacy of L. reuteri S3

**Figure 7:**
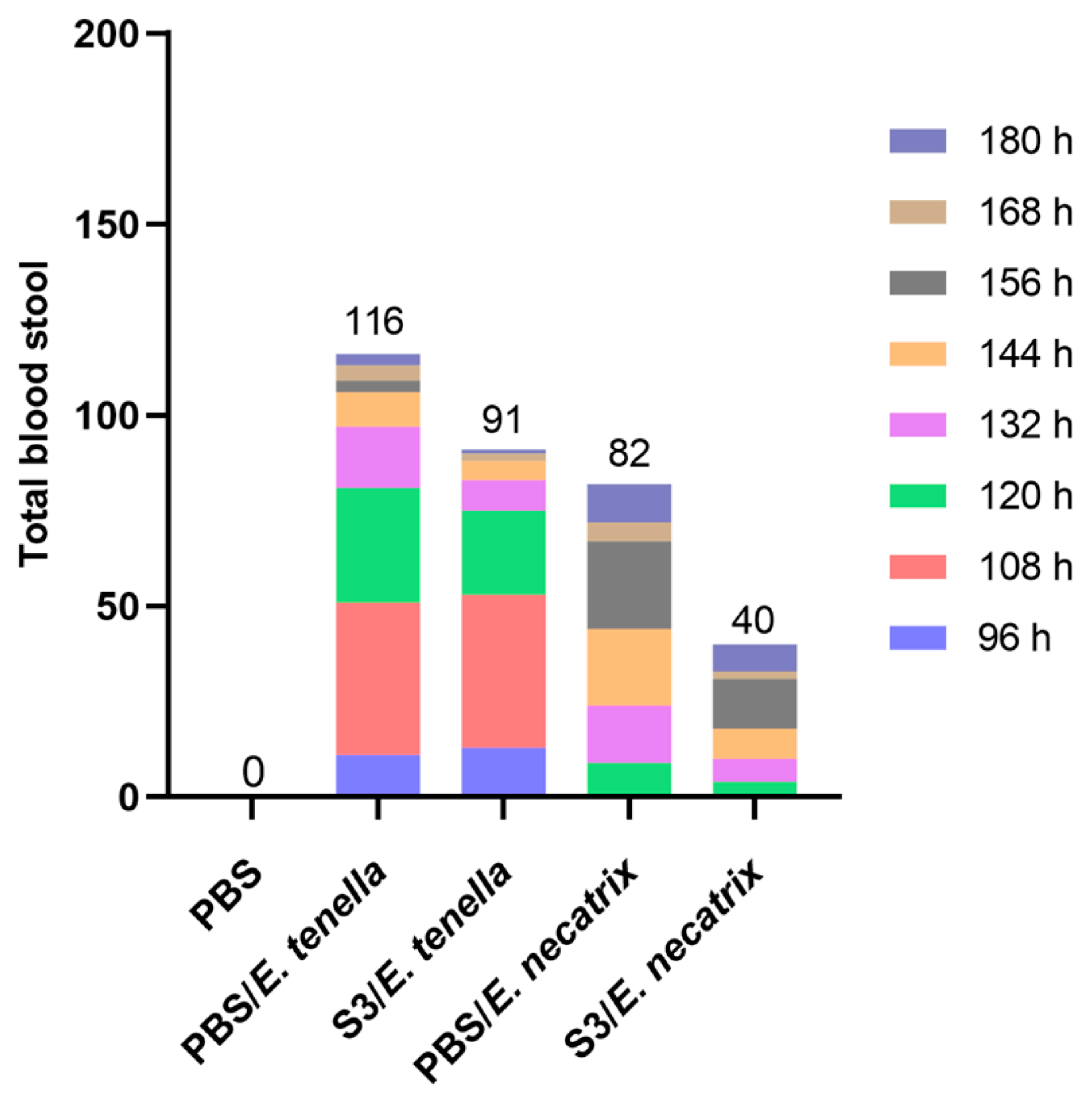
Cumulative number of bloody droppings collected from each treatment group after the onset of bloody diarrhea following *E. tenella* and *E. necatrix* challenge. Bloody droppings were collected every 12 h.

Anticoccidial efficacy is summarized in Table 5. No mortality occurred in any group. The S3/*E. tenella* and S3/*E. necatrix* groups had fewer bloody droppings than the corresponding PBS groups (Figure 7). BWG was significantly lower in the PBS/*E. tenella* and PBS/*E. maxima* groups than the control (*p* < 0.05), while the reduction in the PBS/*E. necatrix* group was not significant (*p* > 0.05). All S3 groups showed partial recovery of BWG relative to their PBS counterparts, with the S3/*E. necatrix* group having the highest RWG. ROP for the S3/*E. tenella*, S3/*E. maxima*, and S3/*E. necatrix* groups were 29.16%, 25.37%, and 28.57%, respectively. Lesion scores were significantly lower in the S3/*E. tenella*, S3/*E. maxima* and S3/*E. necatrix* groups than the corresponding PBS groups (*p* < 0.05). ACI values for the S3/*E. tenella*, S3/*E. maxima*, and S3/*E. necatrix* groups were 130.67, 148.06, and 163.40, respectively, indicating low to moderate anticoccidial efficacy, with the best effect against *E. necatrix* (Table 5).

### 3.8. Peripheral Blood Lymphocyte Subsets

**Figure 8:**
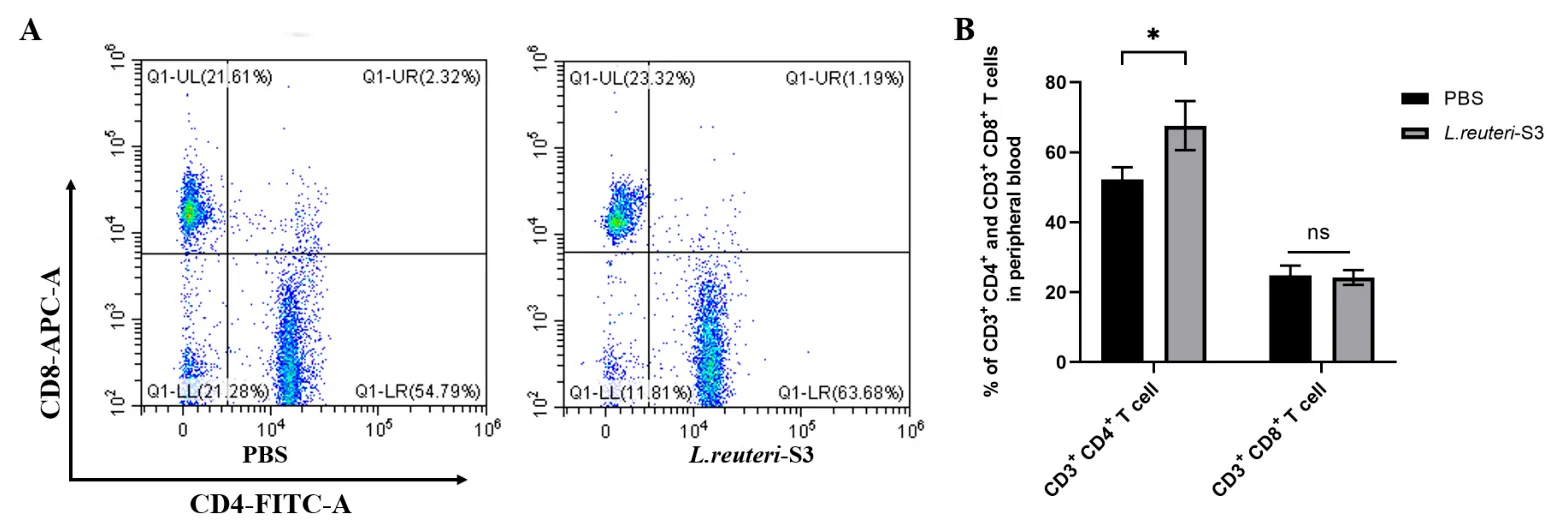
Proportions of CD3^+^CD4^+^ and CD3^+^CD8^+^ T lymphocytes in peripheral blood of Suqin laying hens after *L. reuteri* S3 administration. (**A**) Representative flow cytometry dot plots. (**B**) Summary of flow cytometry results. Data are presented as mean ± SD (*n* = 3 birds per group). Statistical significance was analyzed using Student’s *t*-test. *, *p* < 0.05, and ns, not significant.

Flow cytometry showed that *L. reuteri* S3 administration significantly increased the proportion of CD3^+^CD4^+^ T lymphocytes compared with the control (67.63 ± 7.00% vs. 52.23 ± 3.52%, *p* < 0.05), while CD3^+^CD8^+^ T lymphocyte proportions did not differ between groups (24.78 ± 2.81% vs. 24.22 ± 2.12%, *p* > 0.05) (Figure 8).

## 4. Discussion

*L. reuteri* is a gut symbiont that primarily colonizes in the intestines of humans and different animals, including rodents, pigs, cows, yaks, and chickens [52,53]. Different strains of *L. reuteri* have undergone an evolutionary process through natural selection in different hosts, which has given them special capabilities [31,54,55]. Probiotic strains isolated from the target host species generally exhibit better colonization capacity and ecological adaptation than those from other sources [56,57,58]. In this study, eight *L. reuteri* strains were isolated from White Leghorn chickens. Based on the tested probiotic criteria [59], *L. reuteri* S3, isolated from the jejunum of chickens, was identified as the most promising candidate. The identification of S3 highlights the potential of host-derived *L. reuteri* strains as promising probiotic candidates for avian coccidiosis control, particularly in the context of *Eimeria*-associated gut microbial disturbances.

Survival in the gastrointestinal environment is a prerequisite for oral probiotics to exert their functional effects, given the low pH of gastric juice and the antimicrobial action of bile salts [60,61,62]. Adhesion to the intestinal mucosa, even temporarily, is considered an important criterion for probiotic strain selection [63,64]. While the correlation between in vitro adhesion and in vivo colonization remains to be fully established, strong adhesive properties are generally considered favorable for intestinal colonization [65]. In this study, three isolates (S2, S3, and C3) maintained viability above 50% in simulated gastric fluid, intestinal fluid, and fresh chicken bile, indicating their potential to survive in the gastrointestinal tract. *L. reuteri* S3 showed significantly higher adhesion to DF-1 cells than the two reference strains. In vivo experimental results showed that, in the cecum, *L. reuteri* abundance remained elevated in the *L. reuteri* S3-treated group compared with the control after 18 days of supplementation followed by a 7-day withdrawal, indicating that S3 achieved persistent colonization in the cecum.

The ability to inhibit the growth of pathogens is a favorable characteristic of probiotic bacteria [66]. The absence of antibiotic resistance genes is an important aspect of probiotic product [67]. In the present study, three isolated strains, including S3 strain, could inhibit the growth of *S. pullorum*, *E. coli*, and *S. aureus*, suggesting potential for controlling common poultry pathogens. Antibiotic susceptibility testing revealed that S3 strain was sensitivity to erythromycin, clarithromycin, clindamycin, chloramphenicol, and penicillin, and resistance to vancomycin, ciprofloxacin, ofloxacin, polymyxin B, spectinomycin, nitrofurantoin, oxacillin, co-trimoxazole, tetracycline, and minocycline, and resistance to vancomycin, fluoroquinolones, and co-trimoxazole. This resistance to vancomycin, fluoroquinolones, and co-trimoxazole is consistent with intrinsic resistance patterns reported for lactobacilli, which are typically chromosomally encoded with low risk of horizontal transfer [68,69,70]. Nevertheless, whole-genome sequencing would be necessary prior to commercial application of to confirm the absence of transferable resistance genes. Additionally, it is necessary to determine whether the S3 strain produces antimicrobial substances such as reuterin. Moreover, some *L. reuteri* strains are capable of producing other bioactive metabolites, including exopolysaccharides (EPSs), B-group vitamins (particularly vitamin B12 and folate), short-chain fatty acids (SCFAs), and bioactive peptides, which may contribute to gut homeostasis, immune regulation, and host health [71,72,73]. However, the production of these metabolites is highly strain-specific, and their functional mechanisms remain incompletely understood. Therefore, further studies are warranted to identify and characterize the metabolites produced by strain S3 and to elucidate the molecular mechanisms underlying its probiotic effects.

Several authors have previously reported that probiotic supplementation has a positive effect on the growth performance and serum biochemical parameters of chickens [74,75,76,77,78,79,80,81,82]. In the present study, administering *L. reuteri* S3 to White Leghorn chickens and Suqin laying hens for 18 consecutive days resulted in improved ADG. The effect of administration *L. reuteri* S3 on serum biochemical parameters varied between White Leghorn chicken and Suqin laying hens. Among the seven serum biochemical parameters detected in White Leghorn chickens, only ALT was significantly increased in the high-dose group, suggesting that excessive doses may cause mild hepatic stress, and dose optimization is needed for practical application. In Suqin laying hens, TP and GLO were significantly higher in all S3-treated groups (*p* < 0.05), possibly reflecting enhanced immune function, superior protein digestibility, and improved liver health [79,81]. ALB was significantly increased, and TC was significantly decreased in the high-dose group (*p* < 0.05). An increase in ALB levels serves as a direct biochemical indicator of enhanced nutritional absorption, superior liver function, and a heightened state of protein anabolism [82]. A reduction in TC levels is a widely accepted biochemical indicator strongly correlated with systemic metabolic improvements, enhanced end-product quality, and robust intestinal health [78]. These results implied that *L. reuteri* S3 exhibits greater probiotic potential for Suqin laying hens.

Probiotic supplementation also has a positive effect on the immune, digestive, and metabolic organ indices of chickens [74,76,77,79,81]. In the present study, although there were no significant differences in the liver, spleen, kidney, and Bursa of Fabricius indices between *L. reuteri* S3-treated group and the control group, the spleen and Bursa of Fabricius indices of *L. reuteri* S3-treated group were higher than those of the control group. The thymus index was significantly higher in the low- and medium-dose groups of both breeds. An increase in thymus, spleen, and Bursa of Fabricius index suggests that *L. reuteri* S3 may stimulate cellular proliferation, antibody production, and lymphoid tissue development to boost immunity. Further observation is needed to determine the impact of *L. reuteri* S3 on digestive organs of chickens.

The results of the present study indicated that supplementation with *L. reuteri* S3 could modulate intestinal microbiota composition, with a correlation to the administered dose. The Chao1 and Shannon indices were higher in the medium-dose jejunal and low-dose cecal groups. In the jejunum, the abundance of *L. vaginalis* increased in the low-dose group, while the abundance of *L. johnsonii* increased in the medium-dose group. Both of these bacteria have been reported to enhance intestinal barrier function and modulate immune responses [83,84]. However, in the high-dose group, the abundance of *E. coli* increased, whereas the abundance of *Lactobacillus* decreased. In the cecum, following a 7-day withdrawal period after 18 days of supplementation, *L. reuteri* abundance remained elevated in the medium- and high-dose groups compared with the control, indicating that *L. reuteri* S3 achieved persistent colonization in the cecum. The abundances of *L. crispatus* and *P. dorei* were reduced across all treatment groups, while the abundance of *F. plautii* increased in the low-dose group, reflecting competitive interactions among bacterial species. These findings indicated that supplementation with *L. reuteri* S3 can enhance gut microbial diversity, thereby improving gut stability and host health [85,86], and promote the proliferation and colonization of beneficial lactobacilli. Notably, the administration dose of *L. reuteri* S3 may influence the direction and magnitude of microbiota modulation, and excessive supplementation could disrupt microbial balance. Therefore, dose optimization remains necessary prior to clinical application to achieve beneficial effects. In addition, whether *L. reuteri* S3 exerts similar effects on gut microbiota in Suqin laying hens remains to be confirmed in future studies.

Currently, there is a lack of unified standards for evaluating the efficacy of probiotics against avian coccidiosis. In the present study, following the evaluation methods used for anticoccidial drugs [50,51,87], we employed several indicators to assess the efficacy of probiotics against avian coccidiosis, including number of bloody diarrhea, survival rate, average weight gain and relative body weight gain, oocyst output and oocyst reduction rate, intestinal lesion score, and anticoccidial index. Among these indicators, the number of bloody diarrhea and lesion score reflect the severity of disease manifestation. Average weight gain and relative weight gain indicate the impact of coccidian infection on chicken production performance. Oocyst output and oocyst reduction rate reflect the potential of probiotics to block the development of coccidia. The results showed that supplementation with *L. reuteri* S3 could alleviate clinical symptoms, reduce weight loss, decrease oocyst shedding, and mitigate the severity of intestinal lesions caused by infection with *E. tenella*, *E. maxima*, and *E. necatrix*. The protective effects may involve competitive exclusion at the mucosal surface, reinforcement of epithelial barrier integrity, stimulation of local SIgA production, and microbiota-mediated colonization resistance [88]. However, possibly due to the lack of a direct inhibitory or killing effect on coccidia, *L. reuteri* S3 may not have the potential to directly and effectively prevent coccidia development and oocyst production, resulting in a relatively low ACI values. Moreover, *L. reuteri* S3 exhibited differential anticoccidial efficacy against *E. tenella*, *E. maxima*, and *E. necatrix*, with an ACI value exceeding 160 against *E. necatrix*, indicating moderate anticoccidial activity. Further research is needed to investigate the varying efficacies of *L. reuteri* S3 against these three coccidia species.

A previous research reported that dietary supplementation with *L. reuteri* improved growth performance in *E. tenella*-infected broilers [30]. The selective expansion of CD4^+^ T cells observed prior to challenge may have contributed to the anticoccidial protection, as CD4^+^ T cells play an important role in coordinating immune responses against *Eimeria* infection [89]. The dietary supplementation with *Lactobacillus acidophilus* increased CD4^+^ T lymphocyte proportions in chickens, supporting the immunomodulatory potential of lactobacilli [90]. In the present study, supplementation with *L. reuteri* S3 significantly increased Bursa of Fabricius indices and the proportion of CD3^+^CD4^+^ T cells in Suqin laying hens, while CD3^+^CD8^+^ T cell proportions were unaffected. CD4^+^ T cells coordinate adaptive immune responses through cytokine secretion and activation of immune effector cells [91]. Therefore, the selective expansion of this subset suggests that *L. reuteri* S3 may enhance immune capacity in chickens prior to pathogen challenge. However, the Immunoprotective mechanism explaining why *L. reuteri* S3 is effective against *E. necatrix* but less so against *E. tenella*, *E. maxima* remains to be studied.

## 5. Conclusions

In the present study, we isolated eight *L. reuteri* strains from the jejunum and cecum of White Leghorn chickens in China. These findings highlight the potential of chicken-derived *L. reuteri* as a probiotic candidate for avian coccidiosis control. After comprehensive evaluation of probiotic properties based on established criteria, *L. reuteri* S3 was identified as a promising candidate. *L. reuteri* S3 exhibited differential anticoccidial efficacy against *E. tenella*, *E. maxima*, and *E. necatrix*, with an ACI value exceeding 160 against *E. necatrix*. This suggests that *L. reuteri* S3 exhibits significant potential for utilization in a feed additive for avian coccidiosis control. However, further studies are warranted to fully explore the probiotic potential of strain S3 by identifying and characterizing its secreted metabolites and elucidating the underlying molecular mechanisms.

## Figures and Tables

**Table 1:** Antibacterial activity of *L. reuteri* isolates against three pathogenic bacteria.

Indicator Bacteria	Zone Diameter of Inhibition (mm)		
	*Salmonella pullorum*	*Escherichia coli*	*Staphylococcus aureus*
cLV	10.23 ± 0.58 ^ab^	10.18 ± 0.49 ^a^	11.92 ± 0.69 ^ab^
cLP	8.63 ± 0.65 ^a^	11.35 ±0.57 ^a^	10.60 ± 1.43 ^a^
S2	9.20 ± 1.23 ^a^	10.13 ± 1.27 ^a^	12.07 ± 0.95 ^ab^
S3	11.57 ± 0.82 ^b^	12.10 ± 0.90 ^a^	14.13 ± 0.70 ^bc^
C3	11.77 ± 0.61 ^b^	11.03 ±1.95 ^a^	14.53 ± 0.72 ^c^

Note: Different superscript letters within the same column indicate significant differences (*p* < 0.05).

**Table 2:** Antibiotic susceptibility of *L. reuteri* S3.

Antibiotics	Zone of Inhibition Diameter	Susceptibility
Names		
Erythromycin	22.00 ± 0.31	S
Clarithromycin	23.20 ± 0.25	S
Clindamycin	22.87 ± 0.12	S
Chloramphenicol	27.87 ± 0.16	S
Nitrofurantoin	-	R
Penicillin	23.40 ± 0.36	S
Oxacillin	-	R
Tetracycline	-	R
Minocycline	-	R
Ciprofloxacin	-	R
Ofloxacin	-	R
Vancomycin	-	R
Polymyxin B	-	R
Co-trimoxazole	-	R
Spectinomycin	12.63 ± 0.06	R

Note: R, resistance; S, sensitivity.

**Table 3:** Effects of *L. reuteri* S3 on serum biochemical parameters in White Leghorn chickens.

Items		Groups		
	PBS	S3 Low	S3 Middle	S3 High
ALT (U/L)	2.50 ± 0.46 ^a^	2.47 ± 0.06 ^a^	2.37 ± 0.23 ^a^	3.07 ± 0.15 ^b^
AST (U/L)	267.10 ± 14.24 ^a^	248.00 ± 1.54 ^a^	267.00 ± 23.91 ^a^	257.10 ± 11.98 ^a^
TP (g/L)	23.97 ± 1.76 ^a^	24.83 ± 1.53 ^a^	23.57 ± 1.21 ^a^	25.10 ± 0.75 ^a^
ALB (g/L)	10.03 ± 0.31 ^a^	9.63 ± 0.65 ^a^	9.37 ± 0.25 ^a^	9.97 ± 0.50 ^a^
GLO (g/L)	13.93 ± 1.46 ^a^	15.20 ± 0.98 ^a^	14.20 ± 0.96 ^a^	15.13 ± 0.67 ^a^
A/G (%)	0.72 ± 0.06 ^a^	0.63 ± 0.04 ^a^	0.66 ± 0.03 ^a^	0.66 ± 0.05 ^a^
UREA (mmol/L)	0.55 ± 0.02 ^a^	0.44 ± 0.13 ^a^	0.49 ± 0.06 ^a^	0.60 ± 0.07 ^a^
TC (mmol/L)	3.10 ± 0.19 ^a^	3.11 ± 0.03 ^a^	3.21 ± 0.06 ^a^	2.91 ± 0.31 ^a^
TG (mmol/L)	0.40 ± 0.10 ^a^	0.37 ± 0.04 ^a^	0.43 ± 0.03 ^a^	0.40 ± 0.08 ^a^

Note: Different superscript letters within the same row indicate significant differences (*p* < 0.05). ALT: alanine aminotransferase; AST: aspartate aminotransferase; TP: total protein; ALB: albumin; GLO: globulin; A/G: albumin/globulin ratio; UREA: urea; TC: total cholesterol; TG: triglycerides.

**Table 4:** Effects of *L. reuteri* S3 on serum biochemical parameters in Suqin laying hens.

Items		Groups		
	PBS	S3 Low	S3 Middle	S3 High
ALT (U/L)	2.67 ± 0.06 ^a^	2.90 ± 0.20 ^a^	2.73 ± 0.12 ^a^	2.97 ± 0.25 ^a^
AST (U/L)	241.07 ± 1.88 ^ab^	243.63 ± 3.16 ^b^	235.70 ± 1.42 ^a^	237.37 ± 2.76 ^a^
TP (g/L)	29.03 ± 0.15 ^a^	31.67 ± 0.51 ^b^	31.27 ± 0.31 ^b^	31.87 ± 0.15 ^b^
ALB (g/L)	10.17 ± 0.67 ^a^	11.17 ± 0.50 ^ab^	10.37 ± 0.42 ^ab^	11.40 ± 0.60 ^b^
GLO (g/L)	18.87 ± 0.67 ^a^	20.50 ± 0.17 ^b^	20.90 ± 0.40 ^b^	20.47 ± 0.51 ^b^
A/G (%)	0.54 ± 0.05 ^a^	0.54 ± 0.03 ^a^	0.49 ± 0.03 ^a^	0.56 ± 0.04 ^a^
UREA (mmol/L)	0.37 ± 0.02 ^a^	0.38 ± 0.02 ^a^	0.39 ± 0.03 ^a^	0.40 ± 0.02 ^a^
TC (mmol/L)	3.58 ± 0.03 ^b^	3.60 ± 0.05 ^b^	3.67 ± 0.05 ^b^	3.49 ± 0.06 ^a^
TG (mmol/L)	0.38 ± 0.10 ^a^	0.39 ± 0.02 ^a^	0.40 ± 0.42 ^a^	0.38 ± 0.01 ^a^

Note: Different superscript letters within the same row indicate significant differences (*p* < 0.05). ALT: alanine aminotransferase; AST: aspartate aminotransferase; TP: total protein; ALB: albumin; GLO: globulin; A/G: albumin/globulin ratio; UREA: urea; TC: total cholesterol; TG: triglycerides.

**Table 5:** Protective efficacy of *L. reuteri* S3 against *E. tenella*, *E. maxima*, and *E. necatrix* challenge in Suqin laying hens.

Groups	SR (%)	BWG (g)	RWG (%)	OP/Per Bird (10^8^)	ROP (%)	LS	LI	OI	ACI
PBS/Control	100	77.10 ± 16.04 ^b^	100.00	0.00	100.00	0.00 ± 0.00 ^a^	0.00	0	200.00
PBS/*E. tenella*	100	43.81 ± 26.64 ^a^	56.82	8.95	/	2.55 ± 0.69 ^c^	25.50	40	91.32
S3/*E. tenella*	100	54.10 ± 13.14 ^a^	70.17	6.34	29.16	1.95 ± 0.76 ^b^	19.50	20	130.67
PBS/*E. maxima*	100	53.53 ± 15.17 ^a^	69.43	3.39	/	2.40 ± 0.74 ^c^	24.00	40	105.43
S3/*E. maxima*	100	65.20 ± 20.30 ^ab^	84.56	2.53	25.37	1.65 ± 0.75 ^b^	16.50	20	148.06
PBS/*E. necatrix*	100	61.92 ± 21.14 ^b^	80.32	0.56	/	2.20 ± 0.63 ^c^	22.00	40	118.32
S3/*E. necatrix*	100	72.78 ± 13.23 ^b^	94.40	0.40	28.57	1.10 ± 1.05 ^b^	11.00	20	163.40

Note: Comparisons were made between each *Eimeria* species and the control group, respectively. Different superscript letters within the same column indicate significant differences (*p* < 0.05).

## Data Availability

The 16S rDNA sequencing raw data have been saved in the China National Center for Bioinformation, with the accession code (PRJCA066643). The 16S rRNA gene sequences of the eight *L. reuteri* isolates have been deposited in the GenBank database under accession numbers PZ676512–PZ676519. All data and additional files are available from the corresponding author on reasonable request.
